# Effects of *Spartina alterniflora* Invasion on Soil Quality in Coastal Wetland of Beibu Gulf of South China

**DOI:** 10.1371/journal.pone.0168951

**Published:** 2016-12-30

**Authors:** Daobo Wang, Wei Huang, Ruwen Liang, Fusheng Li

**Affiliations:** 1 Guangxi Key Laboratory of Beibu Gulf Marine Biodiversity Conservation, Guangxi colleges and universities Key Laboratory of Exploitation and Protection of Beibu Gulf Marine Biological Resources, College of Resources and Environment, Qinzhou University, Guangxi, China; 2 College of Agriculture, Guangxi University, Guangxi, China; Shandong University, CHINA

## Abstract

**Background:**

Since *Spartina alterniflora* (simplified as *Spartina*) has strong ecological competitiveness and rapid growth, it has been introduced and living in the coastal wetland regions of China for more than 30 years. Taking coastal wetland in the Beibu Gulf of south China as an example, the effects of *Spartina* invasion on soil quality were investigated to provide scientific basis for soil management.

**Methodology:**

The soil quality of six different coastal wetlands, i.e. mangrove (vegetation coverage is above 95%), mangrove- *Spartina* ecotones (vegetation coverage is above 95%), sparse mangrove (vegetation coverage is 10%-20%), sparse mangrove- *Spartina* ecotones (vegetation coverage is about 80%), *Spartina* (vegetation coverage is about 80%) and bare beach (no plants), were analyzed using the following indicators: pH, cation exchange capacity, contents of total nitrogen, total phosphorus and organic carbon, microbial biomass carbon, microbial biomass nitrogen, microbial carbon / organic carbon, and activities of urease, acid phosphatase, invertase, polyphenol oxidase and catalase.

**Principal Findings:**

The results showed that compared to mangrove wetland, most indicators in the mangrove-*Spartina* wetland showed a decline tendency except pH value, and the contents of total phosphorus and organic carbon, microbial biomass carbon and soil microbial biomass nitrogen, and the activities of acid phosphatase and invertase were significantly reduced (P<0.05). Compared to sparse mangrove wetland and bare beach, the *Spartina* invasion wetland (sparse mangrove-*Spartina* wetland and *Spartina* wetland) had higher contents of total nitrogen, total phosphorus and organic carbon, microbial biomass carbon, microbial biomass nitrogen, cation exchange capacity and the activities of urease and acid phosphatase, so soil quality in the sparse mangrove wetland and bare beach was significantly improved. Factor Analysis and PCA also showed that: the quality of mangrove wetland soil is better than that of mangrove-*Spartina* ecotones wetland soil; the quality of sparse mangrove-*Spartina* ecotones wetland soil is better than that of sparse mangrove wetland soil; the quality of *Spartina* wetland soil is better than that of bare beach wetland soil.

**Conclusions/Significance:**

Therefore, in the invaded Beibu Gulf wetland ecosystems of south China, for the mangrove wetlands where the productivity of native plant was higher than that of *Spartina*, the *Spartina* invasion can cause soil degradation significantly and it must be strictly controlled, while for sparse mangrove wetland and bare beach where the productivity of native plant was lower than that of *Spartina*, *Spartina* invasion can improve the soil quality. Thus our study may help to better understand the effect of plant invasion.

## Introduction

*Spartina alterniflora* (simplified as *Spartina*) is a perennial herb originating from the mud flat on the coast of the Atlantic [1]. *Spartina* not only has the roles of promoting deposition and creating land, making the bare beach green, soil improvement, and beach protection, but also provides biological products, pollutant degradation and environmental purification.

*Spartina* has been introduced and rapidly invaded all Chinese coastal wetlands since 1970s [2–3]. Compared with the local species of Suaeda (*Suaeda salsa*) and Reed (*Phragmites australis*) in the coastal area of the Yangtze River basin, the aboveground and underground biomasses of *Spartina* are five times as much as those of *Suaeda salsa*, and *Spartina* community decreases soil respiration-rate, increases soil organic carbon (SOC), and improves the carbon sequestration capacity [4–5]. The *Spartina* invasion significantly increases the primary productivity and carbon sequestration capacity of the ecosystems. The annual net primary productivity of *Spartina* ecosystem is 21.6 t C/ha, which is higher than that of the native species of reed ecosystem (16.9 t C/ha), and the decomposition rate of litter is less than that of reed ecosystem [6], which results in higher soil SOC content than that of the reed ecosystem. At the same time, *Spartina* promotes soil N accumulation in the wetland, which may further enhance the *Spartina* invasion [7].

However, many studies have shown the contrary conclusions [8–10]. Because *Spartina* grows and spreads quickly on the beach in south China, it becomes a typical alien invasive species, which competes for the resources of mangroves and threatens the mangrove ecosystem. The *Spartina* invasion not only causes severe degradation of mangrove habitat [8], but also changes bio-diversity and behavior pattern of mangrove ecosystem. For example, the density, species diversity and abundance of benthic animals in the *Spartina* invasion areas are higher than those of mangrove areas, but lower than those of the beach area in the Jiulong River estuary mangrove of south China [9]. The *Spartina* invasion also changes the abundance of ammonia oxidizing archaea and bacteria, and influences the community structure of ammonia oxidizing microorganisms [10].

In recent years, there are some studies about the effects of *Spartina* invasion on soil quality of coastal wetland, but the responses of different wetlands to the *Spartina* invasion are not the same. There are various coastal wetlands in Beibu Gulf of south China, such as the mangrove wetland, sparse mangrove wetland and bare beach. Various coastal wetlands have different contents of nutrient and organic carbon and enzyme activities in soils, so their responses to the *Spartina* invasion will not be the same. Thus our hypothesis was that the effects of *Spartina* invasion on soil quality of mangrove wetland, sparse mangrove wetland and bare beach in Beibu Gulf of south China were different, so as to provide the reasonable soil management and ecological resilience of coastal wetland after the *Spartina* invasion.

## Materials and Methods

### Ethics statement

The study was carried out in the National Ocean Park of Qinzhou Maowei Sea Mangrove, but without disturbing the nature reservation, and the field studies did not involve endangered or protected species. The Oceanic Administration of Qinzhou is the authority responsible for Maowei Sea national park. No specific permissions were required for these locations and every one can go to Maowei Sea national park freely.

### Site description

**Fig 1:**
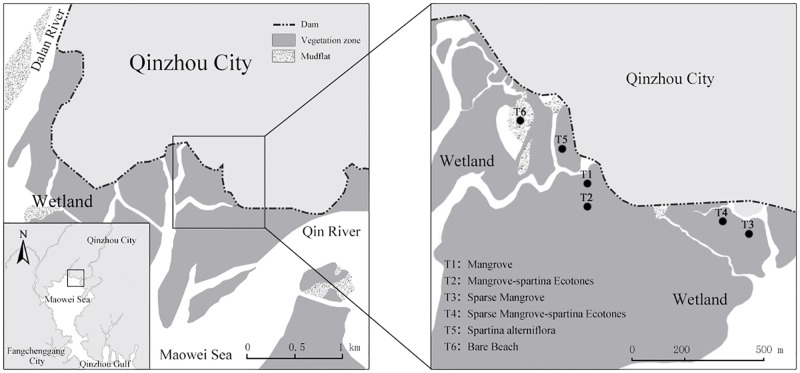
Location of sampling sites in the Maowei Sea National Ocean Park. The study area was drawn using Photoshop software, with remote sensing data downloaded from web (http://map.51240.com/) without copyright restrictions.

The National Ocean Park is located in the Beibu Gulf region of south China (North latitude 21°38'- 21°57', East longitude 108°27'- 108°44') with a total area of 27.84 km^2^ and mangrove area of 18.93 km^2^ (Fig 1). In order to minimize the sampling error, the sampling point is about 100 m away from tidal water level in different plots. The position and distance of sampling points is shown in Fig 1.

There are 11 families and 16 species mangrove plants in the protected area, the main types of community include *Aegiceras corniculatum*, *Avicennia marina*, *Avicennia marina* & *Aegiceras corniculatum*, and *Kandelia candel & Aegiceras corniculatum*. *Kandelia candel*, *Bruguiera gymnorrhiza*, *Rhizophora stylosa*, *Avicennia marina*, *Acanthus* etc. occupies 43.2% of Chinese mangrove species. Among them, *Acanthaceae acanthus* is rare mangrove plants, and *Bruguiera gymnorrhiza* and *Rhizophora stylosa* are both the endangered species.

According to the growth condition of mangrove and *Spartina*, six coastal wetlands can be divided, i.e. mangrove (vegetation coverage is above 95%), mangrove–*Spartina* ecotones (vegetation coverage is above 95%), sparse mangrove (vegetation coverage is 10%-20%), sparse mangrove -*Spartina* ecotones (vegetation coverage is about 80%), *Spartina* (vegetation coverage is about 80%) and bare beach (no plants). Basic data of plant community at different sampling sites was shown in Table 1.

**Table 1:** Basic data of plant community at sampling sites.

Treatment	Wetland	Vegetation	Vegetation coverage	Height (m)		Litter	Soil surface
				Mangrove	*Spartina*		
**T**_**1**_	Mangrove	*Kandelia candel* *Aegiceras coriculatum*	above 95%	3	/	Brown leaf litter	Dark
**T**_**2**_	Mangrove—*Spartina* ecotones	*Spartina alterniflora* *Aegiceras coriculatum*	above 95%	3	2–2.5	Dry branches and fallen leaves	Dark brown with small amount of silt accumulation
**T**_**3**_	Sparse mangrove	*Aegiceras coriculatum*	10%-20%	2	/	Small amount of litter	Greater sand content
**T**_**4**_	Sparse mangrove -*Spartina* ecotones	*Aegiceras coriculatum*	about 80%	2	2	Small amount of litter	More single grain of sand
**T**_**5**_	*Spartina*	*Spartina alterniflora*	about 80%	/	2	Nearly no litter	Starting to development
**T**_**6**_	Bare beach	No plants	No plants	/	/	Little algae floating	Suspended silt

### Soil sampling and measurements

Due to typhoon from June to October, soil sampling was not taken in this period. In this study, three soil samples were taken from each treatment on April 25 2015 and April 27 2016, respectively. Soil samples were taken from 0 to 30 cm layers using stainless steel drill. After picking up the grass roots and other debris, part of soil sample was grinded and packed into bags after 0.15 mm sieves after air-drying. The other part was kept in the refrigerator at 4°C, which was used for the determination of soil enzyme activities, and microbial biomass carbon and nitrogen.

Soil pH was measured at water and soil ratio of 5: 1 using pH meter of Orian 818 type [11]. Soil organic carbon was determined using the potassium dichromate method-external heating method [11]. Total nitrogen and phosphorus contents were determined using Semimicro-Kjeldahl method and molybdate-blue spectrophotometry, respectively [11]. Cation exchange capacity (CEC) was determined using NH_4_AC exchange method [11].

Soil microbial biomass C (N) was determined using chloroform fumigation and K_2_SO_4_ extraction method [12–13]. Soil microbial biomass C (N) = the amount of C (N) in fumigation samples—the amount of C (N) non-fumigation samples. Microbial biomass C = Ec/0.38, and microbial biomass N = E_N_/0.45.

The activities of urease, acid-phosphatase, surcease, polyphenol oxidase and catalase in soils were respectively measured using sodium phenoxide- sodium hypochlorite colorimetric method, disodium phenyl phosphate colorimetric method, 3, 5-dinitrosalicylic acid colorimetric method, pyrogallic acid colorimetric method and potassium hypermanganate titration method [14].

### Statistical method

The mean values were calculated for each parameter. One-way ANOVA (analysis of variance) and the correlation coefficient among different soil indexes was performed using SPSS 17.0 for Windows. Duncan’s multiple-range test was used to compare the mean values in different when ANOVA indicated statistical significance at P<0.05.

There are a lot of indices to evaluate the soil quality. But till now, no index was recognized as the best one. So factor analysis and principal component analysis (PCA) were used to find the main indices to evaluate soil quality.

Factor analysis was performed using SPSS17.0 for Windows. All the measured data were involved in the calculation. In factor analysis, principal component was employed for the extraction method. Extraction was on the basis of eigenvalues, when eigenvalues is greater than 1, maximum iteration for convergence is set to 25 times. The maximum variance (Vari_max_) method was adopted for rotation. Factor scores were selected by regression method and saved as variables.

PCA was performed using SPSS17.0 for Windows. The component matrix data (V) obtained by factor analysis were the primary data for PCA. The weighted coefficient (F_i_)at the i^th^ principal component was calculated as follows:

$$F_{i}=\frac{V}{T_{i}}$$ (1)

where T_i_ is the total variance of initial eigenvalue at the i^th^ component. The weighted method was used to calculate the i^th^ principal component scores of each sample. Duncan’s multiple-range test was used to compare the significance of principal components.

## Results

### Soil nutrient, pH and CEC

Table 2 showed the effect of *Spartina* invasion on soil nutrient, pH and cation exchange capacity (CEC) in different coastal wetlands. The *Spartina* invasion had significant effect on soil nutrient content and its related indices in different coastal wetlands. From the results in 2015 and 2016, the contents of total nitrogen and total phosphorus and CEC were the largest in mangrove wetland (T_1_) and the lowest in bare beach (T_6_).

**Table 2:** Effects of *Spartina* invasion on soil nutrient, pH and cation exchange capacity (CEC) in different coastal wetlands.

Year	Treatment	Total Nitrogen (g/kg)	Total Phosphorus (g/kg)	pH	CEC (cmol/kg)
**2015**	**T**_**1**_	1.973±0.224a	1.154±0.039a	5.58±0.13d	16.348±0.083a
	**T**_**2**_	1.560±0.103ab	0.951±0.075b	5.82±0.10d	16.255±0.079ab
	**T**_**3**_	0.687±0.103d	0.533±0.011c	6.30±0.12b	14.732±0.428c
	**T**_**4**_	1.269±0.178b	0.940±0.033b	6.24±0.06bc	15.666±0.091b
	**T**_**5**_	1.231±0.155b	0.919±0.038b	6.33±0.07b	15.964±0.088ab
	**T**_**6**_	0.491±0.071d	0.429±0.007c	6.82±0.11a	13.396±0.168d
**2016**	**T**_**1**_	1.974±0.218a	1.158±0.044a	5.72±0.14d	16.362±0.070a
	**T**_**2**_	1.570±0.121ab	0.959±0.070b	5.91±0.13cd	16.263±0.085ab
	**T**_**3**_	0.686±0.107d	0.532±0.013c	6.54±0.16ab	14.739±0.428c
	**T**_**4**_	1.260±0.168b	0.946±0.037b	6.39±0.13b	15.668±0.098b
	**T**_**5**_	1.235±0.151b	0.922±0.039b	6.54±0.13ab	15.978±0.090ab
	**T**_**6**_	0.495±0.068d	0.425±0.012c	6.85±0.08a	13.405±0.168d
Significance test					
*F value*		12.513	43.178	12.501	29.385
*P* value		<0.001	<0.001	<0.001	<0.001

Different lowercase letters means that the difference was significant at P_0.05_ level. T_1_: Mangrove, T_2_: Mangrove- *Spartina* ecotones, T_3_: Sparse mangrove, T_4_: Sparse mangrove- *Spartina* ecotones, T_5_: *Spartina*, T_6_: bare beach, and the following was the same.

Compared to T_6_, T_1_, T_2_ (mangrove–*Spartina* wetland), T_4_ (sparse mangrove -*Spartina* wetland) and T_5_ (*Spartina* wetland) increased the total nitrogen content by 301.8%, 217.8%, 158.5% and 150.6% in 2015, and 298.9%, 217.1%, 154.6% and 149.4% in 2016 (P<0.05), respectively (Table 2). The total nitrogen content in T_4_ was 84.7% in 2015 and 83.7% in 2016 higher than that of T_3_ (sparse mangrove wetland) (P<0.05).

Compared with T_1_, T_2_, T_3_, T_4_, T_5_ and T_6_ decreased the total phosphorus content by 17.6%, 53.8%, 18.5%, 20.4% and 62.8% in 2015, and 17.2%, 54.1%, 18.3%, 20.4% and 63.3% in 2016 (P<0.05) (Table 2). Compared to T_3_, T_4_ increased the total phosphorus content by 76.5% in 2015 and 77.8% in 2016 (P<0.05). The total phosphorus content in T_5_ was 114.0% in 2015 and 117.0% in 2016 higher than that of T_6_ (P<0.05).

Compared to T_6_, T_1_, T_2_ and T_4_ decreased soil pH by 18.2%, 14.6% and 8.6% in 2015, and 16.5%, 13.7% and 6.7% in 2016 (P<0.05), and T_3_ and T_5_ decreased soil pH by 7.6% and 7.2% in 2015 (P<0.05), respectively (Table 2).

As shown in Table 2, compared to T_6_, T_1_, T_2_, T_3_, T_4_ and T_5_ increased soil CEC by 22.03%, 21.34%, 9.98%, 16.95% and 19.17% in 2015, and 22.06%, 21.32%, 9.95%, 16.88% and 19.19% in 2016, respectively (P<0.05). But the difference between T_1_ and T_2_ was not significant, and the difference was also not significant among T_2_, T_4_ and T_5_. Compared to T_3_, T_1_, T_2_, T_4_ and T_5_ increased soil CEC by 10.97%, 10.34%, 6.34% and 8.36% in 2015, and 11.01%, 10.34%, 6.30% and 8.40% in 2016, respectively (P<0.05).

### Soil organic carbon, microbial biomass carbon and nitrogen

Table 3 showed the effect of *Spartina* invasion on soil organic carbon, microbial biomass carbon and nitrogen in different coastal wetlands. The *Spartina* invasion had significant effects on the soil organic carbon, microbial biomass carbon and nitrogen.

**Table 3:** Effects of *Spartina* invasion on soil organic carbon, microbial biomass carbon and nitrogen in different coastal wetlands.

Year	Treatment	Organic carbon (C_org_) (g/kg)	Microbial biomass carbon (C_mic_) (mg/kg)	Microbial biomass nitrogen (mg/kg)	C_mic_/C_org_ (%)
**2015**	**T**_**1**_	23.611±1.823a	932.067±53.742a	104.628±6.538a	3.961±0.11a
	**T**_**2**_	19.776±1.113b	720.910±57.032b	81.127±6.524b	3.638±0.10ab
	**T**_**3**_	10.049±0.777d	329.875±27.584d	38.909±3.018c	3.280±0.04b
	**T**_**4**_	16.257±0.682c	557.916±23.300c	66.972±7.818b	3.433±0.04b
	**T**_**5**_	14.992±0.749c	537.143±23.399c	66.542±8.979b	3.585±0.04ab
	**T**_**6**_	8.110±0.345d	231.735±30.980d	25.385±2.160c	2.836±0.26c
**2016**	**T**_**1**_	23.618±1.822a	933.081±53.748a	108.317±7.091a	3.964±0.11a
	**T**_**2**_	19.806±1.132b	724.607±55.538b	85.199±6.113b	3.652±0.09ab
	**T**_**3**_	10.047±0.776d	332.772±28.147d	43.783±2.892c	3.310±0.05b
	**T**_**4**_	16.303±0.709c	565.790±22.620c	71.502±7.882b	3.472±0.04b
	**T**_**5**_	15.020±0.748c	540.967±24.334c	71.515±8.162b	3.604±0.05ab
	**T**_**6**_	8.109±0.346d	233.974±29.884d	31.571±2.128c	2.865±0.24c
Significance test					
*F value*		29.126	40.008	18.573	8.535
*P* value		<0.001	<0.001	<0.001	<0.001

As illustrated in Table 3, T_1_ had the highest content of organic carbon (23.611 g/kg in 2015 and 23.618 g/kg in 2016), and compared to T_1_, T_2_, T_3_, T_4_, T_5_ and T_6_ decreased organic carbon content by 16.2%, 57.4%, 31.1%, 36.5% and 65.7% in 2015, and 16.1%, 57.5%, 31.0%, 36.4% and 65.7% in 2016, respectively (P<0.05). But the difference between T_3_ and T_6_ was not significant, and the difference between T_4_ and T_5_ was not significant, either. Compared to T_3_, T_4_ increased the organic carbon content by 61.8% in 2015 and 62.3% in 2016 (P<0.05). Compared with T_6_, T_5_ increased the organic carbon content by 84.8% in 2015 and 85.2% in 2016 (P<0.05).

The changing trend of the microbial biomass carbon was consistent with that of the organic carbon content. Compared to T_1_, T_2_, T_3_, T_4_, T_5_ and T_6_ decreased the microbial biomass carbon by 22.7%, 64.6%, 40.1%, 42.4% and 75.1% in 2015, and 22.3%, 64.3%, 39.4%, 42.0% and 74.9% in 2016, respectively (P<0.05). But the difference between T_3_ and T_6_ was not significant, and the difference between T_5_ and T_4_ was not significant, either. The microbial biomass carbon in T_4_ was 69.1% in 2015 and 70.0% in 2016 higher than that of T_3_ (P<0.05), and the microbial biomass carbon in T_5_ was 131.8% in 2015 and 131.2% in 2016 higher than that of T_6_ (P<0.05).

Like microbial biomass carbon, the microbial biomass nitrogen is also one of the sensitive indicators in soil quality change [15]. Compared to T_1_, T_2_, T_3_, T_4_, T_5_ and T_6_ decreased the microbial biomass nitrogen by 22.5%, 62.8%, 36.0%, 36.4% and 75.7% in 2015, and 21.3%, 59.6%, 34.0%, 34.0% and 74.9% in 2016, respectively (P<0.05). But the difference between T_3_ and T_6_ was not significant, and the difference among T_2_, T_4_ and T_5_ was not significant, either. The microbial biomass nitrogen in T_4_ was 72.1% in 2015 and 63.3% in 2016 higher than that of T_3_ (P<0.05). Compared with T_6_, T_5_ increased the microbial biomass nitrogen by 162.1% in 2015 and 126.5% in 2016 (P<0.05).

The ratio of microbial biomass carbon to organic carbon (Cmic/Corg) is used as an indicator to reflect the change of soil organic matter [16], and it can predict long-term changes in soil organic matter and monitor land degradation or recovery. Compared with T_1_, T_3_, T_4_ and T_6_ decreased the Cmic/Corg by 17.2%, 13.3% and 28.4% in 2015, and 16.5%, 12.4% and 27.7% in 2016 (P<0.05). But the differences between T_1_ and T_2_, or T_3_ and T_4_ were not significant. C_mic_/C_org_ in T_5_ was 26.4% in 2015 and 25.8% in 2016 higher than that of T_6_ (P<0.05).

### Soil enzyme activity

Effect of *Spartina* invasion on soil enzyme activity in different coastal wetlands was presented in Table 4. The *Spartina* invasion affected soil enzyme activities significantly. Compared with T_6_, T_1_, T_2_, T_4_ and T_5_ increased the urease activity by 78.5%, 58.3%, 46.3% and 46.2% in 2015, and 77.1%, 57.8%, 45.9% and 46.0% in 2016, respectively (P<0.05). But the differences between T_1_, T_2_, T_4_ and T_5_ were not significant. The urease activity in T_4_ was 39.6% in 2015 and 39.3% in 2016 higher than that of T_3_ (P<0.05).

**Table 4:** Effects of *Spartina* invasion on soil enzyme activity in different coastal wetlands.

Year	Treatment	Urease (NH_3_—N mg/g)	Acid Phosphatase (mg/kg)	Invertase (mg/g 24 h)	Polyphenol Oxidase (mg/g)	Catalase (ml 0.02 mol/L KMnO_4_ /g)
**2015**	**T**_**1**_	4.040±0.176a	0.139±0.009a	25.118±2.615a	0.635±0.029a	17.566±0.862a
	**T**_**2**_	3.583±0.320a	0.113±0.003b	17.540±2.621b	0.640±0.029a	16.601±0.511ab
	**T**_**3**_	2.372±0.218b	0.059±0.006d	9.581±0.609c	0.474±0.027b	14.288±0.404cd
	**T**_**4**_	3.310±0.261a	0.086±0.008c	11.925±1.559bc	0.520±0.032b	15.624±0.470bc
	**T**_**5**_	3.309±0.302a	0.083±0.007c	11.727±1.739bc	0.509±0.037b	15.641±0.390bc
	**T**_**6**_	2.263±0.271b	0.043±0.003d	7.936±1.704c	0.425±0.030b	13.252±0.211d
**2016**	**T**_**1**_	4.143±0.188a	0.140±0.010a	25.246±2.632a	0.637±0.031a	17.655±0.878a
	**T**_**2**_	3.691±0.315a	0.114±0.004b	17.630±2.643b	0.643±0.030a	16.685±0.524ab
	**T**_**3**_	2.449±0.249b	0.059±0.007d	9.628±0.622c	0.476±0.028b	14.359±0.408cd
	**T**_**4**_	3.412±0.265a	0.086±0.008c	11.986±1.572bc	0.523±0.033b	15.703±0.479bc
	**T**_**5**_	3.415±0.341a	0.083±0.008c	11.788±1.754bc	0.512±0.038b	15.720±0.399bc
	**T**_**6**_	2.339±0.301b	0.043±0.004d	7.972±1.720c	0.427±0.030b	13.318±0.204d
Significance test						
*F value*		6.110	23.522	9.789	7.011	8.191
*P* value		<0.001	<0.001	<0.001	<0.001	<0.001

T_1_ had the highest activity of acid-phosphatase (0.139 mg/g soil in 2015 and 0.140 mg/g soil in 2016), and T_6_ had the lowest activity (0.043 mg/g soil) (Table 4). The acid-phosphatase activity in T_1_ was 18.5%, 57.8%, 38.1%, 40.3% and 69.3% in 2015, and 18.6%, 57.9%, 38.3%, 40.5% and 69.3% in 2016 higher than that of T_2_, T_3_, T_4_, T_5_ and T_6_ treatments (P<0.05), respectively. Compared with T_3_, T_4_ increased the acid-phosphatase activity by 46.6% in 2015 and 46.3% in 2016 (P<0.05). T_5_ increased the acid-phosphatase activity by 94.5% in 2015 and 93.8% in 2016 (P<0.05) if compared with T_6_.

The effect of *Spartina* invasion on the invertase activity in the mangrove wetland was significant (Table 4). Compared to T_1_, T_2_, T_3_, T_4_, T_5_ and T_6_ decreased the invertase activity by 30.2%, 61.9%, 52.5%, 53.3% and 68.4% in 2015, respectively (P<0.05), and the trend in 2016 was similar to that of 2015. But the difference between T_3_ and T_4_ was not significant, and the difference between T_6_ and T_5_ was not significant, either.

As presented in Table 4, compared to T_1_, T_3_ and T_6_ decreased the polyphenol oxidase activity by 25.32% and 33.09% in 2015, and 25.26% and 33.05% in 2016 (P<0.05), respectively. But there were no significant differences between T_1_ and T_2_, or between T_3_ and T_4_, or between T_5_ and T_6_.

Table 4 showed that T_1_ had the highest activity of soil catalase (17.566 mg/g soil in 2015 and 17.655 mg/g soil in 2016), and T_6_ had the lowest activity (13.252 mg/g soil in 2015 and 13.318 mg/g soil in 2016). Compared to T_6_, T_5_ increased the catalase activity by 18.03% in 2015 and 18.04% in 2016 (P<0.05), respectively.

### Correlation, Factor analysis and principal component analysis

Table 5 showed that the correlation among different soil indexes. It could be seen from Table 5 that the difference in different soil indexes was significant at P_0.01_ level each other. All indicators were positive correlation except for pH value. pH values were negatively correlated with the other indicators.

**Table 5:** The Correlation Coefficient Among Different Soil Indexes. MB-N: Microbial biomass nitrogen. TRAP: Acid Phosphatase. PPO: Polyphenol Oxidase.

	**Total N**	**Total P**	**pH**	**CEC**	**C**_**org**_	**C**_**mic**_	**MB-N**	**C**_**mic**_**/C**_**org**_	**Urease**	**TRAP**	**Invertase**	**PPO**	**Catalase**
**Total N**	1												
**Total P**	0.835*	1											
**pH**	-0.753*	-0.759*	1										
**CEC**	0.820*	0.897*	-0.739*	1									
**C**_**org**_	0.888*	0.882*	-0.828*	0.839*	1								
**C**_**mic**_	0.879*	0.884*	-0.831*	0.837*	0.992*	1							
**MB-N**	0.835*	0.888*	-0.830*	0.850*	0.891*	0.908*	1						
**C**_**mic**_**/C**_**org**_	0.725*	0.801*	-0.720*	0.817*	0.802*	0.854*	0.827*	1					
**Urease**	0.829*	0.865*	-0.648*	0.756*	0.761*	0.758*	0.739*	0.614*	1				
**TRAP**	0.854*	0.870*	-0.855*	0.799*	0.888*	0.904*	0.928*	0.789*	0.857*	1			
**Invertase**	0.757*	0.759*	-0.760*	0.665*	0.765*	0.792*	0.807*	0.646*	0.812*	0.927*	1		
**PPO**	0.756*	0.741*	-0.728*	0.702*	0.745*	0.748*	0.756*	0.612*	0.825*	0.914*	0.894*	1	
**Catalase**	0.717*	0.912*	-0.779*	0.839*	0.829*	0.844*	0.824*	0.783*	0.803*	0.849*	0.821*	0.786*	1

*The difference was significant at P_0.01_ level.

Two statistical methods (Kaiser-Meyer-Olkin and Bartlett’s test of sphericity) were used to perform factor analysis. In Kaiser-Meyer-Olkin (KMO), KMO metric for sampling sufficient degree was 0.784, and Bartlett’s test of sphericity showed extremely significant difference (P<0.001), the selected factor is suitable.

From Table 6, the communalities of extracted value, was very high (>0.75), indicating that most of the information can be extracted by factor analysis. So the results of factor analysis were effective and credible, and all the measured data can be used as the original data to perform factor analysis.

**Table 6:** Statistics of factor analysis. Extraction method: principal component analysis. Rotation method: vari_max_ with Kaiser normalization. Orthogonal rotation method: Kaiser.

	Communalities		Component matrix	Rotated component matrix
	Initial	Extract	1	1
**pH**	1	0.753	-0.867	-0.087
**Organic carbon**	1	0.887	0.942	0.094
**Total nitrogen**	1	0.820	0.906	0.090
**Total phosphorus**	1	0.883	0.940	0.094
**CEC**	1	0.791	0.889	0.089
**Microbial biomass carbon**	1	0.899	0.948	0.095
**Microbial biomass nitrogen**	1	0.878	0.937	0.094
**Invertase activity**	1	0.792	0.890	0.089
**Urease activity**	1	0.774	0.880	0.088
**Acid phosphatase activity**	1	0.944	0.971	0.097
**Catalase activity**	1	0.833	0.913	0.091
**Polyphenol oxidase activity**	1	0.764	0.874	0.087

**Fig 2:**
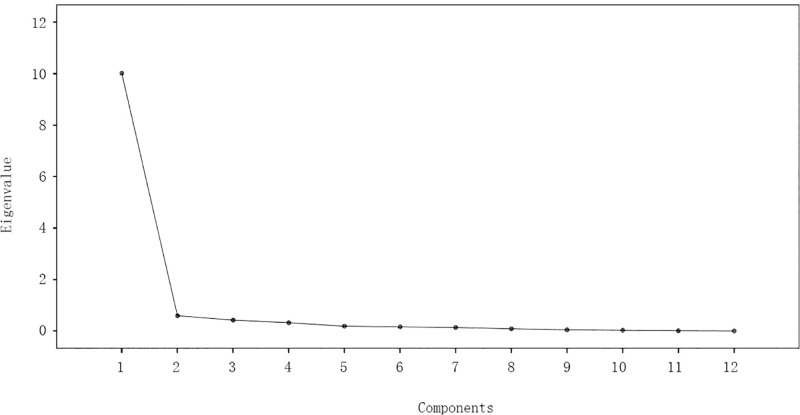
Scree plot of factor analysis.

Table 7 showed that only the factor characteristic value of the first principal component was more than 1, which was 10.018 and accounted for 83.48% of the variance. From Fig 2, the slope between the first and the second principal components was great, but the slope of the rest was gradually gentle. From Table 7 and Fig 2, only one principal component was selected.

**Table 7:** Total variance explained by factor analysis. Extraction method: principal component analysis.

Component	Initial Eigenvalues			Extraction sum of squared loading		
	Total	% of variance	Cumulative (%)	Total	% of variance	Cumulative (%)
**1**	10.018	83.481	83.481	10.018	83.481	83.481
**2**	0.592	4.932	88.413			
**3**	0.421	3.509	91.922			
**4**	0.320	2.668	94.590			
**5**	0.185	1.541	96.131			
**6**	0.160	1.331	97.462			
**7**	0.134	1.121	98.583			
**8**	0.085	0.710	99.293			
**9**	0.047	0.388	99.680			
**10**	0.026	0.218	99.899			
**11**	0.010	0.080	99.978			
**12**	0.003	0.022	100.000			

According to the feature vector matrix and formula (1), the calculation formula for principal component was obtained as follows

$${\text{Z}}_{1}=-0.274{\text{X}}_{1}+0.298{\text{X}}_{2}+0.286{\text{X}}_{3}+0.297{\text{X}}_{4}+0.281{\text{X}}_{5}+0.3{\text{X}}_{6}+0.296{\text{X}}_{7}+0.281{\text{X}}_{8}+0.278{\text{X}}_{9}+0.307{\text{X}}_{10}+0.288{\text{X}}_{11}+0.276{\text{X}}_{12}$$

**Fig 3:**
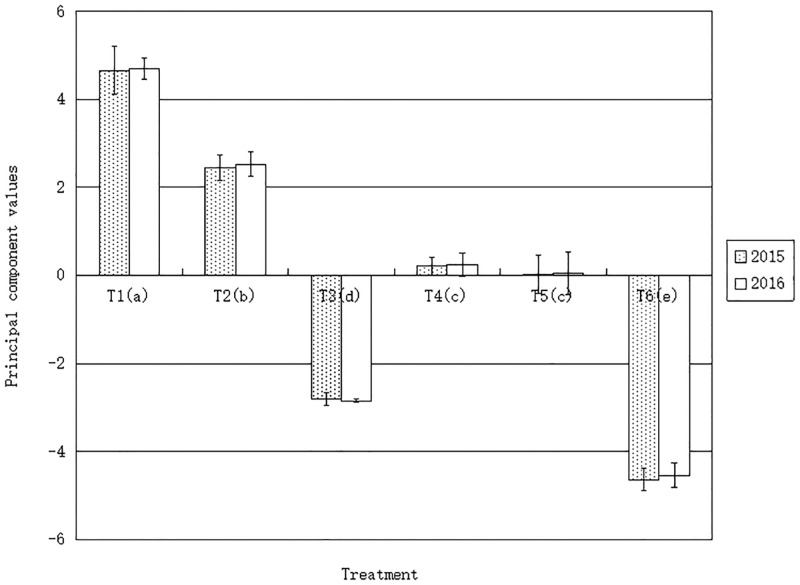
Principal component value. Different letters in brackets indicated the difference at P<0.05 level. The same letter indicates that the difference is not significant. And the following was the same.

The results of the principal component analysis were shown in Fig 3. As only one principal component was selected, the value of the first principal component can basically be used to evaluate the soil quality. From Fig 3, for each treatment, no significant difference was found between the two years. The order of the principal components of each treatment was: T_1_>T_2_>T_4_>T_5_>T_3_>T_6_. There was no significant difference between T_4_ and T_5_, but the differences between the other treatments were significant.

## Discussion

### Soil nutrient, pH and CEC

Unlike the biomass and productivity of the native species, the soil nutrient content is easily changed by the alien plants [17]. Previous researches showed that the *Spartina* invasion increases the accumulation of the total nitrogen content in the wetland soil [7, 18], which in turn enhances the growth rate and biomass of *Spartina* [19]. Because the *Spartina* invasion increases root biomass and *Spartina* can absorb nitrogen form that cannot be absorbed by native plants, the increase of aboveground net primary production provides more energy for nitrogen fixing microorganisms, and thus the content of total nitrogen is improved in the coastal wetland [20]. The accumulation of total organic carbon, total nitrogen and total phosphorus in *Spartina* wetland was far greater than that of other *Artemisia halodendron* and reed wetland in intertidal flat wetland [4]. Nitrogen pool in *Spartina* community was higher than that of the native plants, such as *Scirpus mariqueter* and *Phragmites australis*, and soil nitrogen cycle can be altered by the *Spartina* invasion significantly [21–22]. The results of these studies are not completely consistent with the results of this study. This study showed that the *Spartina* invasion increased soil total N content in the sparse mangrove wetland and bare beach significantly, but reduced it in the mangrove wetland. The possible reason is that litter N content of the mangrove wetland was significantly greater than that of *Spartina* wetland. Zhang et al. (2008) also showed that the *Spartina* invasion decreased soil nitrogen and phosphorus contents in the mangrove wetland [8]. In the relevant literatures, there are no reports on the effect of the *Spartina* invasion on the soil nutrient of the sparse mangrove wetland.

Sundareshwar et al. indicated that the bacterial communities in the wetland soil are limited by phosphorus, and the fixation, storage and release of carbon are affected by the phosphorus limitation of nitrogen transformation bacteria, which indirectly affects the plant production [23]. After adding P, the *Spartina* productivity is significantly higher than that of only nitrogen addition [24]. Therefore, phosphorus is one of the limiting factors for plant productivity in coastal wetland. Previous studies showed that the total phosphorus content and the microbial activity in the bare beach and herbaceous plants wetland are increased by the *Spartina* invasion [25]. Total phosphorus content of reed-*Spartina* ecotones is greater than that of the reed wetland [26]. The accumulation of total nitrogen and phosphorus in *Spartina* is far greater than that of the *Artemisia halodendron* and reed [4]. In this study, the effect of the *Spartina* invasion on soil total P content in coastal wetland was consistent with that of total N content. This is mainly caused by that the *Spartina* productivity is greater than that of native plant in the coastal wetland in previous study, but lower than that of the mangrove wetland.

Soil pH is one of the important attributes in the soil, and it is also an important factor affecting soil fertility. Pan et al. showed that soil pH is significantly increased after the *Spartina* invasion and continued to increase with the increase year of the *Spartina* invasion [27]. The possible reason is that there is surplus NH_4_^+^ in soil as *Spartina* prefers NO_3_^-^. The increase trend of soil pH is also proved by *Berberis thunbergii* [28], *Mikania micrantha* [29] and *Solidago canadensis* [30]. However, there is also evidence that the soil pH in coastal wetland is significantly reduced after the *Spartina* invasion [31]. The possible reason is that H^+^ is produced by the decomposition of soil organic matter with the increase of vegetation decay.

Soil CEC has not been used as the evaluation index of soil quality, but it has been paid more and more attention as it can comprehensively reflect soil fertility, fertilizer retention capacity and buffering capacity [32]. Zhao et al. showed that soil CEC in *Spartina* community was significantly higher than that of other communities [33], but Zhang et al. hold the opposite opinion [8]. In this study, the *Spartina* invasion increased soil CEC in the sparse mangrove wetland and bare beach significantly, but had no significant change in the mangrove wetland. Soil CEC decreases with the degradation of wetland ecosystem, and it can also be used as an important indicator whether wetland ecosystem is degradation or not [34]. From this point of view, the *Spartina* invasion had no serious degradation of mangrove ecosystem, and can promote the development of bare beach or sparse mangrove ecosystem.

### Soil organic carbon, microbial biomass carbon and nitrogen

The *Spartina* invasion influences carbon sequestration in coastal wetland significantly, which is influenced by the native plants, local climate, soil enzyme and soil nutrient and structure. Compared with the native plants of *Suaeda salsa*, the *Spartina* invasion increases the soil organic carbon (SOC) content by 27.0%-69.6%, and increases ecosystem primary productivity and carbon sequestration capacity significantly [35]. But Bu et al. indicated that *Spartina* invasion does not change SOC significantly [36]. The *Spartina* invasion increases carbon reserves in the rhizosphere soil of reed wetland [37]. The bare beach and the reed community can emission greenhouse gases, while *Spartina* community can absorb greenhouse gases, which may slow the warming trend and increase the content of SOC [38]. Underground biomass of *Spartina* is huge, so larger root quantity contributes to the organic carbon flux [39].

Because the net primary productivity of *Spartina* was greater than that of *Suaeda Salsas*, reed and bare beach, and the decomposition rate of the litter is relatively small, the annual quantity of fixed organic carbon can reach 23.9 Gg in coastal wetland of *Spartina* [40]. In this study, the *Spartina* invasion increased soil organic carbon significantly in the bare beach and sparse mangrove wetland, but reduced it in the mangrove wetland. This is because the mangrove plants have more biomass to return more quantity of litter into soil [41]. It is well known that mangrove wetland has maintained high SOC [42], which is three times as much as the average carbon density of the natural soil [43], but the organic carbon content in the *Spartina* wetland cannot reach this level [44].

With the rapid growth of *Spartina* in coastal intertidal zone, different carbon sources of soil microbes can improve soil physical and chemical properties, microbial activity and microbial biomass [45–46]. The density of aboveground population significantly increases the amount of underground microbial communities [47–49]. Compared with the mangrove, *Spartina* has relatively smaller aboveground biomass and the decay. Thus the microbial biomass in wetland soil is decreased because *Spartina* competes with mangrove for limited resources.

C_mic_/C_org_ can be used to indicate the balance, accumulation or consumption of soil carbon [50]. As influenced by soil type, vegetation coverage, management measures and sampling time, the difference in C_mic_/C_org_ is relatively large [51]. In general, the measures to promote sustainable use of soil, such as increasing straw application, can increase C_mic_/C_org_ [52]. Zhang et al. indicated that active organic carbon and total organic carbon content of *Spartina* wetland are higher than those of *Artemisia halodendron* and other wetlands, and the C_mic_/C_org_ decreases with the growth of *Spartina*, which indicated the competitive declining [53–54]. This is consistent with the results of this study. But it is well known that the productivity of mangrove ecosystem is greater than that of *Spartina*, so the C_mic_/C_org_ was reduced after the *Spartina* invasion into mangrove communities in this study.

### Soil enzyme activity

Soil enzyme activity can reflect the relative intensity of biochemical process in soil [55], so it is an important index to evaluate the soil quality. However, various enzymes in soil have different functions. Soil urease can decompose urea, and its activity can characterize the status of soil nitrogen nutrition [56]. As the pioneer plants, *Spartina* can grow near sea, and often has the habitats of extremely harsh bare mudflats where are usually very poor and subject to high salt stress in soil. In addition, drying *Spartina* may provide more organic matter through a large amount of dry matter to affect soil enzyme activity [45]. Huang et al. concluded that soil organic matter is the key factor regulating soil enzyme activity [57]. Owing to its huge biomass and densities, *Spartina* displayed the greatest potential for carbon input, thus enhancing the enzyme activity and facilitating nutrient cycling in the region of coastal marsh [57]. The results of this study also prove these conclusions. After the *Spartina* invasion, the plant growth in the sparse mangrove wetland and bare beach was significantly promoted to improve the soil enzyme activity in this study.

The enzymes are mainly adsorbed on the soil particles. Mangrove vegetation has significantly higher soil particle retention capacity than *Spartina* vegetation because it can retain fine soil particles due to its large density and stem stout.

### Factor analysis and PCA

Duraisami et al. [58] indicated that PCA can reduce the redundancy degree of soil attribute data sets, and the data of principal component can meet the need to integrate soil information for soil quality assessment. Because the measurement time is suitable and the differences between the indices in different treatments are obvious, the correlation between the indices was relatively high. Based on PCA, one principal component extracted can be used for assessing soil quality.

Factor analysis and PCA showed that the soil quality of mangrove wetland was better than that of mangrove-*Spartina* wetland, while the soil quality of sparse mangrove-*Spartina* wetland was better than that of sparse mangrove wetland and the soil quality of *Spartina* wetland was better than that of bare beach. The results basically reflect the difference of soil quality in different treatments.

## Conclusions

In the invaded Beibu Gulf wetland ecosystems of south China, for coastal wetlands such as mangroves where the productivity of native plant was higher than that of *Spartina*, *Spartina* invasion can significantly decrease soil nutrient content, organic carbon content, microbial carbon, microbial nitrogen and enzyme activity. So it must be strictly controlled. But for coastal wetlands such as sparse mangrove or where the productivity of native plant was lower than that of *Spartina*, even bare beach, the *Spartina* invasion can significantly improve soil quality and increase the soil nutrient content, organic carbon content, microbial carbon, microbial nitrogen and enzyme activity, and the results may help relevant region to better understand the effect of plant invasion.

## Supporting Information

S1 File Original data of this paper. (XLS)
